# Clinical review of the clinical necessity of lumbar punctures performed on adults at National District Hospital Emergency Department

**DOI:** 10.4102/safp.v64i1.5435

**Published:** 2022-08-19

**Authors:** Suné Geldenhuys, Cecil Boltman, Wilhelm J. Steinberg, Johan Botes, Cornel van Rooyen

**Affiliations:** 1 Department of Family Medicine, Faculty of Health Sciences, School of Clinical Medicine, University of the Free State, Bloemfontein, South Africa; 2 Department of Biostatistics, Faculty of Health Sciences, School of Biomedical Sciences, University of the Free State, Bloemfontein, South Africa

**Keywords:** lumbar puncture, district hospital, prevalence, clinically indicated, necessity

## Abstract

**Background:**

Previous studies have found that indications for lumbar punctures (LPs) are managed differently, which raises the question of whether all LPs performed are clinically necessary. This study aimed to determine whether unnecessary (clinically not indicated) LPs were being performed at a district hospital in the Free State, South Africa.

**Method:**

This was a retrospective descriptive study. A list from the National Health Laboratory Service (NHLS) was used to identify all patients on whom an LP was performed in the adult emergency department of National District Hospital (NDH) in Bloemfontein, from 1 January 2018 to 30 June 2018. Data were captured on a data sheet and included demographic information, clinical signs and symptoms the patients presented with and the cerebrospinal fluid results.

**Results:**

A total of 364 patients fit the inclusion criteria. Of these patients, 97 files (26.6%) could not be found, patient gender and LP results could be retrieved from the NHLS barcodes. After reviewing the presenting symptoms and signs captured on the 267 files, the primary researcher considered 150 (56.4%) of the LPs performed to have been carried out unnecessarily. From the total population of 364 patients, 246 (67.6%) of the LP results were normal. Only 118 (32.4%) of the LPs performed showed some form of central nervous system pathology. Of the 150 LPs assessed to have been unnecessarily performed, 124 (84.0%) were normal.

**Conclusion:**

This retrospective review indicates that a high percentage of LPs that were clinically not indicated were performed at NDH during the study period.

## Introduction

Lumbar puncture (LP) was first performed in 1891 by Heinrich Iraneus Quincke on children with tuberculosis (TB) meningitis.^1^ This technique became essential in the diagnosis of different infectious and non-infectious conditions affecting the neurological system and for malignant, demyelinating conditions, therapeutic purposes and anaesthetic procedures.^1,2,3^ The procedure, however, was associated with high mortality because of a lack of imaging techniques. With the introduction of computed tomographic (CT) imaging, the risk of lethal complications from LP secondary to brain lesions could be identified and avoided.^4^

Lumbar punctures and cerebrospinal fluid (CSF) analysis are the cornerstones of diagnosing central nervous system (CNS) infection but are associated with potential complications and require the patient to stay in the emergency unit for an extended period.^5,6^ Every day, thousands of LPs are performed worldwide although the prevalence and indications are not known because of a lack of research.^2^ These data are essential to provide information about appropriate management of CNS diseases and pathology, especially with the increasing burden of the human immunodeficiency virus (HIV).^7^ Although the number of definite indications for LPs has decreased with better neuroimaging, an urgent LP is indicated with suspected CNS infection.^1^ An LP may be the only way to confirm the diagnosis of meningitis, subarachnoid haemorrhage and other neuroinflammatory diseases.^4^

Bacterial meningitis is a potentially life-threatening disease associated with 10% – 30% mortality despite adequate antibiotic treatment.^5,8^ In association with HIV, cryptococcal meningitis accounts for 12% – 44% of mortality.^9^ Even though viral meningitis is more prevalent, bacterial meningitis is associated with high morbidity and mortality. It is also challenging to differentiate clinically between the different forms of meningitis and other CNS diseases that can cause similar meningitis-like symptoms. For these reasons, patients presenting with meningeal signs require urgent LPs and immediate antibiotic treatment. Without proper investigations, patients will be treated inappropriately, and other diagnoses might be missed.^6,8,10^

Lumbar punctures should only be performed after a patient has had a thorough neurological examination.^1,4,11^ Moreover, it is important to exclude any possible existing contraindications, both neurological and non-neurological. Signs indicative of a cerebral mass lesion or raised intracranial pressure, also known as ‘red flags’, include focal neurological symptoms, altered mental status, papilledema and new-onset seizures.^5^ There is a potential risk of brain herniation following the LP, which can result in death if an LP is carried out in the presence of these signs and symptoms.^5,12^ In one South African study, it was found that 53.3% of patients known with HIV, presenting with new-onset seizures, had a space-occupying lesion.^12^ It is recommended to do appropriate CT imaging in these patients before an LP is performed. This, however, is not always possible because of a lack of CT scanners in many South African hospitals. At hospitals where CT scanners are not available, patients are referred to a different facility for CT imaging.^11,12^ This, in turn, can delay diagnoses and treatment or influence CSF analysis if an LP is performed after antibiotic administration. Clinical signs should guide the clinician on whether a CT scan is indicated, as immediate LP is advantageous when there are no signs of a space-occupying lesion and an indication for an LP is present.^12^

With the increasing incidence of meningitis because of the HIV and TB burden in South Africa, it is important to do urgent investigations and treat these patients appropriately.^9,13^ In the emergency department of National District Hospital (NDH), LPs are performed almost daily. Sometimes it can be very challenging to decide whether an LP is indicated, as many of these patients present with non-specific symptoms, and CT imaging is not readily available.

This study aimed to determine whether unnecessary, clinically not indicated LPs were being performed at a district hospital in the Free State province, South Africa.

The objectives of this study were to:

- determine the number of LPs performed at the adult emergency department of NDH in Bloemfontein
- identify the presenting signs and symptoms of patients on whom LPs had been performed
- determine the percentage of LPs of which the results signified pathology
- retrospectively review the appropriateness of LPs performed based on the presenting signs and symptoms
- retrospectively review whether the CSF findings were interpreted correctly by the attending doctor

## Materials and methods

### Study design, population and sampling

This was a retrospective descriptive study. The study population included all the adult patients who received an LP in the emergency department of NDH from 1 January 2018 to 30 June 2018. National District Hospital is a primary health care facility in Bloemfontein, Free State. The hospital has a 24-h emergency department, receiving patients from local clinics and private doctors in Bloemfontein and the surrounding rural towns.

Only patients 18 years and older were considered, as there is a separate emergency department for paediatric patients. A list from the National Health Laboratory Service (NHLS) was used to identify all patients from the NDH emergency department from whom CSF specimens were obtained for analyses. This list was used to trace the patients’ files.

Patients whose files were missing were still included in the analysis in the total amount of LPs carried out, as the LP date, patient gender and the LP results were available from the specimen barcode obtained from NHLS. Any missing information from patients’ files was documented as such.

### Data collection

A data collection form was used to capture relevant information from patient files. Demographic data included gender, age and HIV status, symptoms recorded from the history taken at presentation (fever, neck stiffness, headache, altered mental status or confusion, first onset seizures, vomiting^1,13^ and ‘other’) were captured. Results from the examination (fever, pulse rate, Glasgow Coma Score [GCS], neck stiffness, focal neurology and ‘other’) and serum glucose test were also recorded. Information about the LP included the LP data and the level of the doctor who performed the LP, opening pressure and CSF findings.

A retrospective review was included to decide whether the LP was necessary based on the neurological presenting symptoms and signs. This was a clinical judgement based on the acumen of the primary researcher before reviewing the CSF findings. Further review was made on whether the attending doctor interpreted the CSF results correctly (yes, no or inconclusive).

Using the list provided by the NHLS, a study number was assigned to each patient in order of appearance. This number was recorded on the data collection form to trace patients, should some information be missing.

### Pilot study

A pilot study was performed on 1 November 2019. The first 10 data forms were completed from patient files in the specific study population. All 10 files were included in the study as no changes were made to the data form.

### Data analysis

Results from the data form were transferred onto an Excel spreadsheet. Data were analysed by the Department of Biostatistics, Faculty of Health Sciences at the University of the Free State (UFS), using Statistical Analyses Software (SAS 9.4). Numerical variables were summarised by medians, minimum, maximum or percentiles and categorical variables by frequencies and percentages. Differences between groups were evaluated using appropriate statistical tests (chi-square or Fisher’s exact test) for unpaired data.

The CSF results were interpreted using guidelines from Table 1^14^. Mixed meningitis was defined as a case where a patient had both bacterial meningitis and TB meningitis.

**TABLE 1:** Interpretation of cerebrospinal fluid findings in meningitis.

Findings	Pressure (mmH_2_O)	Glucose (mmol/L)	Protein (g/L)	White blood cell count (cells/mm³)
Normal	80–200	60% of blood glucose level	0.15–0.40	< 5 (lymphocytes)
Bacterial	Increased	Markedly decreased	Markedly increased	Increased often > 1000 (> 80% polys)
Viral	Normal	Normal	Increased	Increased (lymphocytes)
Tuberculosis	Increased	Decreased	Markedly increased	Increased (lymphocytes)
Fungal	Normal – increased	Normal	Usually normal	< 300 (lymphocytes) CLAT positive

*Source:* Kloeck WG, editor. A guide to the management of common medical emergencies in adults. 11th ed. Johannesburg: Academy of Advanced Life Support; 2017.

CLAT, cryptococcus antigen latex test.

### Ethical considerations

The protocol was approved by the Health Sciences Research Ethics Committee of the UFS (UFS-HSD2019/1056/2708) and the Free State Department of Health. The NHLS gave the authorisation to use the patient results, and permission to conduct the study was obtained from NDH’s management. No identifiable information was included on the data form and all data were handled confidentially.

## Results

A total of 364 patients who fit the inclusion criteria were identified. Of these patients, 97 (26.6%) patient files could not be found. With the information from the barcodes received by NHLS, it was possible to obtain results on the date, gender, age and LP result for those patients. The remaining 267 (73.4%) patient files were found for which a data form was completed.

### Demographics

In the total study population of 364, 173 (47.5%) patients were female and 191 (52.5%) were male. Of the 267 available patient files, 132 (49.4%) patients were female and 135 (50.6%) were male. The age of the patients (*n* = 364) ranged between 18 and 92 years, with a median age of 41 years.

The majority of the patients (70.4%, 188 of 267) were HIV-positive, 12.0% (*n* = 32) were HIV-negative whilst the HIV status of 17.6% (*n* = 47) was unknown.

### Presenting signs and symptoms

**FIGURE 1:**
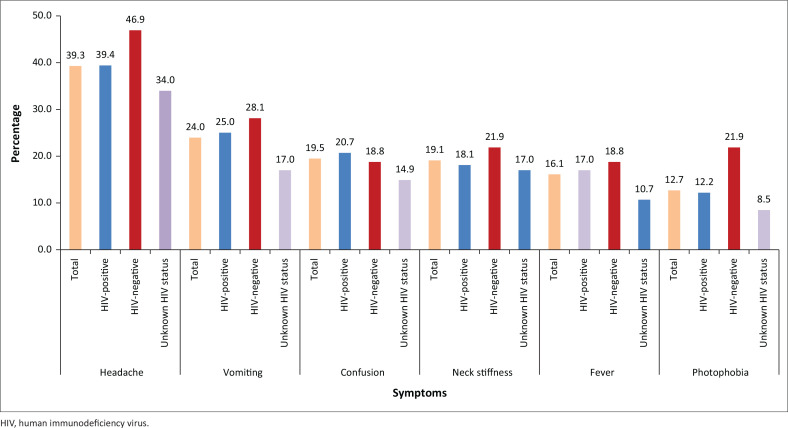
Presenting symptoms in all patients versus HIV-positive, HIV-negative and patients with unknown HIV status.

Presenting symptoms were recorded in all of the 267 files that were retrieved. Patients presented with a wide variety of symptoms, of which headache (39.3%) was the most common. This was followed by vomiting (24.0%), confusion (19.5%) and neck stiffness (19.1%). General body weakness (21.7%) and cough (13.1%) were symptoms most reported under ‘Other’ as summarised in Table 2. Figure 1 illustrates the presenting symptoms in all the patients versus HIV-positive and HIV-negative patients and patients with HIV status unknown.

**TABLE 2:** Frequency of other symptoms in all patients and in patients who are HIV-positive.

Other presenting symptoms	All patients (*n* = 267)		HIV-positive (*n* = 188)	
	*n*	%	*n*	%
General body weakness	58	21.7	52	27.7
Cough	35	13.1	31	16.5
Acute gastroenteritis	25	9.4	22	11.7
Abnormal behaviour	24	9.0	17	9.0
Convulsions – previous episodes	21	7.9	8	4.3
Hallucinations and/or delusions	21	7.9	16	8.5
Loss of appetite	19	7.1	15	8.0
Dizziness	16	6.0	8	4.3
Sweating	14	5.2	12	6.4
Shortness of breath	9	3.4	7	3.7
Cryptococcal antigen positive	6	2.3	6	3.2
Alcohol intoxication and/or history	6	2.3	2	1.1
Chest pain	6	2.3	6	3.2
Blurred vision	5	1.9	4	2.1
Unable to walk	4	1.5	2	1.1
Abdominal pain	4	1.5	3	1.6
Foam from mouth	2	0.8	1	0.5
Muscle spasms	1	0.4	1	0.5
Speech difficulty	1	0.4	0	0.0
Overdose	1	0.4	1	0.5
Asymmetrical weakness	1	0.4	0	0.0
Rapid plasma reagin (RPR) positive	1	0.4	1	0.5
Swollen painful leg	1	0.4	0	0.0

HIV, human immunodeficiency virus.

Similar results were seen for patients who were HIV-positive: 39.4% presented with headache, 27.7% with general body weakness and 25.0% with vomiting. Other common symptoms with HIV-positive patients were coughing (16.5%) and acute gastroenteritis (11.7%). Of the 32 HIV-negative patients, 46.9% (*n* = 15) presented with headaches, followed by 28.1% (*n* = 9) with vomiting. Of the 47 patients with unknown HIV status, 34.0% (*n* = 16) presented with headache, followed by 21.3% (*n* = 10) with neck stiffness.

Except for general body weakness (*p* = 0.0007), cough (*p* = 0.0399) and convulsions – first episode (*p* = 0.003), there were no statistically significant differences between the three groups (HIV-positive, HIV-negative and HIV status unknown) and the presenting symptoms.

### Vital signs and findings during examination

Of the 253 patient files with temperature recorded, 71.5% (*n* = 181) had a normal temperature (35.0 °C – 37.5 °C) on presentation. Only 24.9% (*n* = 63) of the patients had a fever (> 37.5 °C) at the time of their examination. The median temperature was 36.7°C (range: 30.0 °C – 40.5 °C).

Pulse rates were recorded for 266 patients. Almost half of the patients (48.9%, *n* = 130) had a normal pulse on presentation whilst 47.0% (*n* = 125) had tachycardia (> 100 beats per min) and 4.1% (*n* = 11) had bradycardia (< 60 beats per min). The median pulse was 97 beats per min (range: 46–170 beats per min).

Glasgow coma scores were recorded for 184 patients. The majority of patients (56.0%, *n* = 103) had a GCS of 15/15, followed by a GCS of 14/15 in 29.4% (*n* = 54). Only five patients (2.7%) had a GCS of 10 and less. The median GCS was 15 (range: 3–15).

In 76 files (27.5%), no mention of neck stiffness as a sign was made. Of the remaining 191 files, neck stiffness was found on examination in 55.0% (*n* = 105) of the patients and in 42.4% (*n* = 81), it was reported as not present. In five (2.6%) cases, the examining doctor was unsure whether the sign of neck stiffness was positive or not.

Additional neurological signs were observed for 19 patients: asymmetrical decrease in power (36.8%), decrease power globally (26.3%), Brudzinski and/or Kernig (15.8%), postictal (5.3%), dilated pupils (5.3%), constricted pupils (5.3%) and asymmetrical pupils (5.3%).

No serum glucose results were recorded in 37.1% (*n* = 99) of the patients’ files. At the time of the procedure, 4 (1.5%) patients had a finger-prick level > 11 mmol/L, in 8 (3.0%), the glucose result was < 3.6 mmol/L, and in 156 (58.1%), the serum glucose level was recorded as normal.

One patient (1.1%) had focal neurology at the time of examination, whereas in 98.9% (*n* = 87), no signs of focal neurology were reported. The presence of focal neurology was not recorded in 179 files.

### Lumbar puncture results

The opening pressure was only recorded for 5.6% (*n* = 15) of the 267 LPs performed. No pressure was recorded for the remaining 94.4% (*n* = 252) of cases.

Of the total study population of 364 patients on whom LPs were performed, 67.6% (*n* = 246) were classified as normal, which included 47.6% (*n* = 117) female patients and 52.4% (*n* = 129) male patients. Only 32.1% (*n* = 117) of the LPs performed had some form of meningitis and/or CNS pathology whilst the result for one (0.3%) patient was inconclusive. This group included 47.5% (*n* = 56) female patients and 52.5% (*n* = 62) male patients. Of the 188 HIV-positive patients, 62.2% (*n* = 117) of the LPs were normal. Table 3 summarises the LP results.

**TABLE 3:** Lumbar puncture results of total study population and patients who are HIV-positive.

Results	All patients (*n* = 118)		HIV-positive (*n* = 71)	
	*n*	%	*n*	%
Viral meningitis	28	23.7	16	22.5
Mixed meningitis	25	21.2	17	23.9
Cryptococcal meningitis	21	17.8	17	23.9
Bacterial meningitis	19	16.1	7	9.9
TB meningitis	18	15.3	10	14.1
Other CNS pathology	6	5.1	3	4.2
Inconclusive	1	0.9	1	1.4

HIV, human immunodeficiency virus; TB, tuberculosis; CNS, central nervous system.

In the total study population, the highest percentage of patients had viral meningitis (23.7%) and mixed meningitis (21.2%). In the HIV-positive patients, the highest percentage of patients had mixed meningitis and cryptococcal meningitis at 23.9% each.

### Appropriateness of lumbar puncture

After reviewing just the presenting symptoms and signs as captured on the 267 data forms completed, the researcher considered 56.4% (*n* = 150) of the performed LPs were not clinically appropriate. A total of 116 LPs (43.6%) were assessed as clinically indicated. Of the 116 LPs assessed to be appropriate on clinical grounds, 43.1% (*n* = 50) were normal, whilst 56.9% (*n* = 66) tested positive for meningitis. For one patient, the clinical appropriateness was mistakenly not recorded on the data sheet prior to the primary researcher reviewing the CSF findings. It was not possible to assess the appropriateness of indications for the 97 patient files not found, as no information on the presenting symptoms and signs was available. Similarly, 52.1% (*n* = 98) of LPs performed on patients with HIV were considered not clinically appropriate, whilst 47.9% (*n* = 90) were assessed as appropriately indicated.

A total of 126 (84.0%) of the 150 LPs deemed inappropriate were normal. However, 24 (16.0%) patients with LPs deemed inappropriate did have a form of meningitis. Of these patients, 11 (45.8%) had viral meningitis, 4 (16.7%) mixed meningitis, 3 (12.5%) cryptococcal meningitis, 2 (8.3%) bacterial meningitis and 1 (4.2%) TB meningitis. The remaining three patients (12.5%) had a diagnosis of other CNS pathology.

### Level of doctors

Most of the LPs were performed by medical officers (32.2%) and community service doctors (28.4%), followed by interns (24.3%) and registrars in Family Medicine (15.0%). Of the 65 LPs performed by intern doctors, 61.5% (*n* = 40) were assessed as not clinically indicated. A total of 76 LPs were carried out by community service doctors, of which 65.8% (*n* = 50) were assessed as not clinically indicated. These percentages were lower in medical officers and registrars where 44 of 86 LPs (51.2%) and 16 of 40 LPs (40.0%) were found not clinically indicated. There was no statistically significant difference between the level of doctors (*p* = 0.2776).

### Interpretation of cerebrospinal fluid findings

The diagnosis made by the attending doctor after the LP results (*n* = 267) were available were reviewed by the primary researcher using the guidelines (Table 1). The results were interpreted correctly in 96.3% (*n* = 257) of cases. In seven patients (2.6%), the LP results differed from the diagnosis made by the attending doctor. Whether or not the results were correctly interpreted was inconclusive in three (1.1%) cases. For the HIV-positive patients, the results were correctly interpreted in 97.3% (*n* = 183) of cases.

## Discussion

Thousands of LPs are performed worldwide every day although the prevalence and indications are poorly documented because of a lack of research.^2^ Awareness of the indications can increase the proportion of appropriately performed LPs and decrease the total number of LPs.^2^

This research’s concept developed when the primary researcher realised that doctors at NDH are performing a lot of LPs – sometimes it seemed unnecessary. To determine whether the procedure is being used excessively, an audit over a specific period was conducted by extracting data from patient files where LPs for adults were requested. The purpose was to inspect the recorded clinical information and make an expert decision according to the available data on whether the procedure was necessary in each case.

The primary researcher reviewed the available information on the indication for an LP for each patient. All symptoms in the clinical notes were considered, and the expert decision was documented. The LPs’ results were also captured to compare with the primary researcher’s conclusion.

If only considering the clinical presentation reported for the 267 patients, the primary researcher retrospectively assessed that more than half of the LPs performed (56.4%) at NDH were performed unnecessarily. Of those LPs retrospectively reviewed to be clinically not indicated, the outcome of 84.0% was normal. A total of 24 patients, however, did turn out to have a form of meningitis. Of these, 10 patients were diagnosed with a serious form of meningitis (bacterial, TB, mixed or cryptococcus), which can be life threatening if not treated. One can argue that it is better to do an unnecessary LP than to miss a life-threatening case. However, some of these patients might have had a contraindication for an LP and would have needed a CT scan before an LP could safely be performed. It is therefore essential to consider the clinical picture together with the risk and benefits of an LP and perform all necessary investigations.

Interestingly, of the 21 patients who presented with hallucinations, only two patients had meningitis (one viral and one TB meningitis). However, both these patients had other signs and symptoms of meningitis, including headache, neck stiffness, vomiting, dizziness, confusion and tachycardia. A frequent cause of friction experienced by the researchers in the emergency department is the requirement to conduct an LP on all psychiatric patients. Although most of these patients present with some signs, such as hallucination or delirium, the necessary indications to justify LPs are not always present. Of all the LPs conducted for psychiatric patients, only a few are finally diagnosed with clinical meningitis. This is a point for future discussions.

Of the total population of 364 patients on whom LPs were performed, 67.6% of the results were normal. Even though this study could not measure the direct indications for LPs (as not recorded in patient files), the primary researcher’s review as to the appropriateness of the LP was considered. Of the 43.6% of the LPs assessed to be appropriate according to presenting symptoms and the low percentage of LPs (32.4%) that revealed CSF pathology, it is fair to assume that a high percentage of LPs that were not clinically indicated were performed at NDH during this study period. This is likely to be explained by doctors not being fully aware of the indications for LPs, inexperience or because of additional LPs as part of delirium work-up and/or psychiatric work-up that is performed. A study^2^ carried out at two different hospitals in France found that several indications for LPs are managed differently, which raises the question of whether facilities should determine whether doctors are doing too many or too few LPs and if they should consider interventions to modify the numbers of LPs performed.

Studies have highlighted the increasing incidence of meningitis because of the burden of HIV and TB in South Africa, the high mortality associated with it and the importance of doing an urgent investigation and treating these patients appropriately.^9,13^ Most patients in this study (70.4%) were HIV-positive, whilst 12.0% were HIV-negative. In 2019, the HIV prevalence in the general population in South Africa was 20.4%.^15^ The high prevalence of HIV-positive patients might be because doctors are more likely to do an LP in an HIV-positive patient as part of an infective work-up. These patients are also more prone to present with an atypical clinical picture that makes the exclusion of meningitis difficult.

Almost half (46.9%) of the HIV-negative patients presented with headaches compared with 39.4% in HIV-positive patients and 34.0% in patients with unknown status. For vomiting, this occurred in 28.1% of HIV-negative, 25.0% in HIV-positive patients and 17.0% of patients with unknown status. Only 21.9% of HIV-negative patients, 18.1% of HIV-positive patients and 21.3% with unknown status complained of neck stiffness. The history of first onset convulsions was also seen more in HIV-negative patients (21.9%) than in HIV-positive (9.0%) and patients with unknown HIV status (10.7%). These results show a smaller percentage in all of the ‘typical’ symptoms in HIV-positive patients compared with HIV-negative patients. The HIV-positive patients also had a higher percentage combination of ‘other’ symptoms (69.7%) in comparison with the HIV-negative patients (53.1%). This might explain why HIV-positive patients with meningitis seem to have more atypical presentations.

Forty-four percent of patients on whom an LP was performed had a GCS of less than 15. The treating doctor may have used this as an indication to do an LP and might also be a reason why an LP was performed in the absence of many other expected symptoms or signs. A GCS less than 10 was recorded in two patients, which is one of the contraindications to do an LP.^11,13^ One patient presented with focal neurology (focal neurology was recorded for only 88 patients in this study) and seven patients with an asymmetrical decrease in power that are also regarded as contraindications to perform an LP. This means that in 10 (3.6%) of 267 patients, an LP was performed despite a contraindication being present. No mention of a CT scan before the LP was made in these cases. This raises questions as to whether doctors are considering contraindications when performing LPs. Five of these LPs were performed by intern doctors, three by community service doctors and two by medical officers. One can argue that the level of experience played a role; however, at NDH, the interns must discuss all their patients with a senior doctor. It is not clear whether such discussion happened and what nature these discussions entailed.

A serum and/or finger-prick glucose test must be performed at the time of the LP to decide whether the CSF glucose is normal (i.e., 60% of the serum glucose) or abnormal. Patients with abnormal CSF glucose might be hypoglycaemic (3.0% of patients) or hyperglycaemic (1.5% of patients). The CSF glucose should be interpreted in concurrence with the serum glucose to ensure the reliability of the finding. No serum glucose test was carried out in 37.1% of patients that had an LP at NDH. This can lead to misinterpretation of the results and an incorrect diagnosis.

The opening pressure should be recorded to assist with diagnosing the type of meningitis as the pressure is usually raised in bacterial, TB and fungal meningitis. It is also used in repeat LPs performed on cryptococcal meningitis to assess changes in pressure. The opening pressure was only recorded in 5.6% of LPs performed in this study sample. This might be because of a lack of time in a busy emergency department and/or uncooperative patients that make this procedure difficult to do. This, however, is an important omission of LPs performed at NDH.

The majority of the LPs were performed by less experienced clinicians. The relatively low percentage of LPs carried out by the registrars can be attributed to the smaller number of registrars allocated at NDH, but it might also be because of more experience by these doctors who have been working there for a longer duration of time. Almost 70% of LPs performed by community service doctors (65.8%) were deemed unnecessary compared with the registrars’ 40.0%. Clearly those with more clinical experience tend to order less unnecessary LPs. The question arises whether it is safer to do unnecessary LPs when the clinician is inexperienced. We suggest that these clinicians consult a senior doctor with appropriate clinical acumen to assist in the decision-making.

The primary researcher reviewed the diagnosis made by the attending doctor after the LP results were available. For the interpretation of the results, in 2.6% of the patients, the diagnosis made by the attending doctor based on the LP results differed from the diagnosis made by the primary researcher. Even though the researchers assumed that doctors would know how to interpret CSF results, this finding shows that this is not the case. This is concerning as an incorrect diagnosis will lead to the incorrect management and treatment of the patient that can lead to serious morbidity and even mortality.

Another issue that has to be mentioned is the importance of doing a serum or finger-prick glucose test and determining opening pressures, as this would make the interpretation of the CSF findings more reliable.

### Limitations

Not all the files from the study sample could be found. In 97 cases, no review could be made because no clinical information was available because of the missing patient files. The review was only performed by the primary researcher and was not verified by further assessors.

Data on the number of complications post LP, such as post-spinal headache, were not collected. Some information about the examination was not recorded, and in several files, no neurological examination was recorded in the patient notes. It is unknown whether the attending doctor did not mention a sign or symptom because it was not there or did not look for it. As a result of this, the percentages from the different examinations might not reflect the true picture.

## Conclusion

At NDH, LPs are performed almost daily although the indications are not always clear. During the study period, 364 recorded LPs were performed. Common presenting symptoms included headaches, vomiting and neck stiffness. Most patients who received LPs were HIV-positive, which may indicate that clinicians have increased suspicion of infection in these cases. Only a third of these LPs revealed pathology. After reviewing the presenting symptoms and signs, the primary researcher considered 56.4% of the conducted LPs clinically inappropriate. Bearing this in mind, more LPs are being performed at NDH than are necessary. Clear guidelines for emergency rooms outlining the indications for LPs should be available in the form of a protocol to avoid unnecessary LPs and ensure that the patients who need the investigation will get one.^2,7^

### Recommendations

An infective work-up should be individualised to each patient as LP is an invasive procedure that can lead to severe complications.^16^ An LP should not routinely be used in the work-up of psychotic patients without any other evidence of meningitis, especially in known psychiatric patients.

A clear protocol for performing LPs at NDH’s emergency department should be formulated to increase the appropriateness of the LP, especially regarding HIV-positive patients. Training meetings and continuing professional development (CPD) activities are highly recommended to keep practitioners up to date.

An intermittent audit would ensure that the standard operating procedures regarding LPs are performed correctly, including finger-prick glucose test and opening pressure.

Doctors should make comprehensive notes on the CNS examination when performing an LP. In the event of complications and inadequate notes, doctors would expose themselves and their facilities to avoidable medico-legal risk.
